# Effects of moderate- and high-intensity exercise on behavior and oxidative damage in male and female rats subjected to early life stress

**DOI:** 10.1007/s00221-026-07290-1

**Published:** 2026-04-27

**Authors:** Lucas C. Pedro, Rafahel F. Carvalho, Larissa R. Maciel, Nicoly S. Martinello, Flávia S. Niero, Josimar G. Pereira, Catharina de Bem Ribeiro, Lucineia G. Danielski, Fernanda F. Gava, Fabricia Petronilho, Luciano A. da Silva, Gislaine Z. Réus

**Affiliations:** 1 Translational Psychiatry Laboratory, Graduate Program in Health Sciences, University of Southern Santa Catarina (UNESC), Criciuma, SC 88806-000 Brazil; 2 Laboratory of Experimental Neurology, Graduate Program in Health Sciences, Health Sciences Unit, University of Southern Santa Catarina, Criciuma, SC Brazil; 3 Laboratory of Exercise Psychophysiology, Advanced Aquatic Exercise Research Group, University of Southern Santa Catarina (UNESC), Criciúma, Brazil

**Keywords:** Anhedonia, Memory, Anxiety, Exercise, Oxidative stress, Maternal deprivation, Major depressive disorder

## Abstract

Childhood trauma is associated with the development of psychiatric disorders in adulthood, including major depressive disorder (MDD). In preclinical research, animal models are widely used to investigate behavioral and neurobiological alterations related to depressive-like phenotypes, including anhedonia, anxiety-like behavior, and cognitive deficits. Several studies have shown that physical exercise is beneficial for MDD. However, the best type of exercise and intensity is still uncertain. Thus, the present study aimed to investigate the effects of different physical exercise protocols on behavioral outcomes and oxidative damage in rats subjected to maternal deprivation (MD). Moderate or intense aquatic exercise was applied to male and female rats subjected to MD. Anhedonia, anxiety-like behavior, and habituation memory were assessed. In the serum, oxidative stress parameters were analyzed in the prefrontal cortex (PFC) and the hippocampus. MD induced anhedonic-like behavior in females. Moderate aquatic exercise and intense aquatic exercise reduced anxiety in females, MD increased anxious behavior in males, and intense aquatic exercise reversed this effect. In memory, MD induced a deficit in females in the deprived group and males and females with both exercises. MD induces oxidative damage in the brain in a gender-dependent manner, which is generally attenuated by physical exercise. MD induces behavioral changes and increases oxidative damage in adulthood, and exercise protocols may interfere depending on gender and brain region. Further studies are needed to identify the ideal exercise protocol for changes resulting from childhood trauma.

## Introduction

Major depressive disorder (MDD) is among the most common and serious health problems in the world (Batista and de Oliveira 2015). MDD currently affects a total of 322 million worldwide and can also occur in children and adolescents (Silva et al. 2024; Whiteford et al. 2013). The pathophysiology of MDD encompasses several biological mechanisms involving the central nervous system (CNS) and other physiological systems (Verduijn et al. 2015).

Stressful events during early life, encompassing early traumatic experiences, have been shown to induce depressive- and anxiety-like behaviors, as well as cognitive deficits, in rodent models (Carlessi et al. 2022; Giridharan et al. 2019). A study using maternal deprivation (MD) in rats indicates that exposure to adverse events during early development can increase stress reactivity and induce neural circuit alterations that persist into adulthood (Reus et al. 2015). Key mechanisms of MDD include neuroendocrine and inflammatory changes, with a significant role played by the accumulation of reactive oxygen species (ROS) (de Azevedo Cardoso et al. 2023; Ebrahimnejad et al. 2022; Maletic et al. 2007; Réus et al. 2023; Verduijn et al. 2015).

ROS are highly reactive molecules that cause oxidative damage to cellular components, including lipids, proteins, and DNA (Chiang et al. 2018). An imbalance between ROS production and antioxidant capacity leads to oxidative stress, a condition closely associated with the pathophysiology and progression of MDD (Luca and Luca 2019). Furthermore, previous research indicates that oxidative damage is significantly higher in individuals with depression (Adam et al. 2016; Moore et al. 1999; da Silva et al. 2019).

Despite the availability of several antidepressant classes, their limitations (such as delayed onset of action, side effects, and low remission rates) highlight the need for complementary therapeutic strategies, including physical exercise (Kishi et al. 2023; Tsugiyama et al. 2023; Gujral et al. 2017). While the precise mechanisms underlying the antidepressant effects of physical exercise are still being elucidated, evidence suggests the involvement of pathways related to inflammation, oxidative stress, and neural regeneration (Kandola et al. 2019; Schuch et al. 2016).

Physical exercise is widely recognized as an effective adjunct strategy for improving mental health. Evidence suggests that physical activity can enhance psychological well-being and cognitive function, particularly in vulnerable populations (Yang et al. 2022). Additionally, exercise has been shown to significantly reduce anxiety symptoms through combined biological and psychological mechanisms (Kandola and Stubbs 2020). In this context, aquatic exercise has also emerged as a promising approach, with moderate-to-high intensities capable of promoting beneficial brain adaptations (Jackson et al. 2022).

In this regard, exercise modulates brain metabolism and structure, influencing systems involved in learning and memory (Cotman and Berchtold 2007; Kirk-Sanchez and McGough 2014). These benefits are often mediated by reduced inflammation and limited ROS accumulation (Ebrahimnejad et al. 2022). However, defining the appropriate intensity and type of exercise is clinically crucial, as inadequate or overly intense protocols may be ineffective or even pathogenic (Larsen and Matchkov 2016). For instance, inappropriate physical activity can exacerbate oxidative stress and suppress neurogenesis, potentially neutralizing cognitive improvements (Lan et al. 2018). In this context, physical exercise-induced reduction in ROS production may enhance antioxidant repair mechanisms, specifically modulating protein carbonylation (Adam et al. 2016; Moore et al. 1999; da Silva et al. 2019).

Despite the known benefits of exercise, the most effective intensity for mitigating MDD symptoms remains unclear. This study aims to evaluate behavioral effects and oxidative damage following moderate- and intense aquatic exercise protocols in Wistar rats subjected to maternal deprivation (MD). We hypothesize that both exercise intensities can attenuate MD-induced depressive and anxious behaviors and reduce oxidative damage, with these effects depending on the exercise intensity and the specific brain region analyzed.

## Materials and methods

### Animals

Female Wistar rats at three months of age and weighing 250–280 g were obtained from the breeding colony of the University and were housed for one week in the presence of males for mating purposes. At the end of seven days, the pregnant rats were housed individually with *ad libitum* access to food and water until the pups were born and identified. All mothers and pups were maintained on a 12-hour light/dark cycle (06:00 a.m. to 06:00 p.m.) at 23 ± 1 °C. On postnatal day (PND) 1, the MD protocol was applied to a percentage of male (*n* = 36) and female (*n* = 36) pups from days 1–10 PND (deprived, N total = 72); other males (*n* = 12) and females (*n* = 12) were used as controls (non-deprived N total = 24) (Carlessi et al. 2022). All experimental procedures involving rats were performed in accordance with the NIH Guide for the Care and Use of Laboratory Animals and the Brazilian Society for Neuroscience and Behavior recommendations for animal care. The ethics committee approved the experimental protocol under protocol number 59/2021.

### Maternal deprivation (MD)

The deprivation protocol consisted of removing the mother from the residence box and taking her to another room. The pups were maintained in their home cage (grouped in the nest in the presence of maternal odor). The pups were deprived of their mother for three hours a day for the first ten days, while being kept at a temperature of 23 ± 1 °C. This protocol was chosen because it does not require manipulating the pups (Ignácio et al. 2017a, b; Réus et al. 2017). At the end of each daily deprivation session, the mothers were returned to their home boxes; this procedure was performed during the light phase of the cycle, between 8:00 a.m. and 12:00 p.m. The control rats (non-deprived) remained in their resident boxes with their mothers throughout the experiment. Throughout the MD protocol, the bed was changed every 2 days per the facility’s control protocols.

### Physical exercise protocol

On the 21st postnatal day (PND), after weaning, rats from the MD and control groups were housed 4 per cage (males and females kept separate).

Moderate aquatic exercise or intense aquatic exercise protocols were applied to rats subjected to the MD protocol, performed in the morning during the light phase of the cycle. The groups were: (1) controls (no maternal deprivation and no exercise; *n* = 24; 12 females and 12 males); (2) Maternal deprivation (*n* = 24; 12 females and 12 males); (3) Maternal deprivation + Moderate aquatic exercise (*n* = 24; 12 females and 12 males); and (4) Maternal deprivation + Intense aquatic exercise (*n* = 24; 12 females and 12 males). The exercise protocols were applied for 30 days to evaluate the intervention in adulthood. The exercises were performed in water tanks with appropriate dimensions (minimum height of 60 cm and minimum diameter of 30 cm), maintaining the water temperature between 23 °C and 25 °C. A load was attached to each rat’s tail and adjusted individually at the beginning of the week according to body mass. The rats were weighed before the start of the protocol. The rats in the moderate protocol swam continuously, with week 1 (20 min duration and 3% load), week 2 (28 min); week 3 (28 min), and week 4 (28 min). For the rats in the intense protocol, a high-intensity, short-duration interval-training model (advanced aquatic exercise) was employed. In this exercise model, the rats swam for 60 s interspersed with 60 s of rest, using a 1:1 ratio. The rats swam intermittently, from week 1 to 4 (5 sets of 60s x 60s, totaling 10 min and a load of 7%).

Regarding load selection, previous studies indicate that the lactate threshold is reached when supporting a load of 5–6% of body mass (Gobatto et al. 2001). Therefore, the moderate protocol was performed with a progressive load of 3 to 6% of the individual body mass (below the lactate threshold), and the intense protocol was used with an incremental load between 7% and 12% of the rats’ body mass (above the lactate threshold). Exercise training protocols were adapted from previous protocols (Gharaat et al. 2019; Winter et al. 2018). Both groups underwent three training days without the load to adjust to the environment. The animals were continuously monitored throughout all sessions to ensure active engagement in swimming; all animals completed the proposed time for each stage without persistent fluctuations or refusal to swim; therefore, no exclusion criteria were necessary. The 30-min swimming protocol is consistent with previously published studies and has proven to be well tolerated by rodents (Nonato et al. 2016; Stone et al. 2015).

### Behavioral tests

The behavioral tests were performed after exercise protocols on PND 51. All behavior tests were conducted during the light part of the cycle.

#### Habituation memory on the open field test

The habituation to the open field test assesses motor performance during the training session and non-associative memory during the retention session. This apparatus consists of a brown plywood arena measuring 45 × 60 cm, surrounded by wooden walls 50 cm high and containing a front glass wall. The floor of the open field is divided by black lines into nine rectangles (15 × 20 cm each). The rats were gently placed in the left rear quadrant and left to explore the arena for 5 min (training session). Twenty-four hours later (PND 52), they underwent a similar open-field test session (PND 51). Any crossing (frequency with which the rat crossed one of the grid lines with all four paws) and rearing (frequency with which the rat stood on their hind legs in the maze) performed in both sessions were counted (Brown et al. 1999). The decrease in the number of crossings and rearings between the two sessions across all experimental groups was used to measure habituation retention (Réus et al. 2023; Vianna et al. 2000).

#### Anhedonia on the splash test

This test of self-care and motivational behavior involves squirting a 10% sucrose solution onto the dorsal coats of rats. The sucrose solution soiled the rat’s fur and elicited grooming behavior (involving cleaning movements such as licking or washing the face and trunk). The time spent grooming was measured for 5 min, on PND 52, as an index of self-care and motivational behavior (Abelaira et al. 2022; Isingrini et al. 2010).

#### Anxiety on the elevated plus maze test

The elevated plus maze apparatus was made of wood and consisted of two open arms (50 × 10 × 2 cm) and two closed arms (50 × 10 × 40 cm), all facing a central platform (10 × 10 cm) elevated 45 cm above the floor. Rats from all groups on PND 53 were placed in the center of an elevated plus maze facing one of the closed arms. During a 5-min test in a dark room illuminated with red light, the number of entries into each arm and the time spent in each arm were recorded (Gomes et al. 2011; Réus et al. 2023).

### Oxidative stress parameters

After the behavioral tests were complete, the rats were killed by decapitation, and the blood was collected and placed in microcentrifuge tubes. Serum aliquots were obtained from the collected blood by centrifugation at 10,000 RPM for 10 min. In addition, the skulls were removed, then the whole brain was collected and placed on a filter paper and a Petri dish on ice. The prefrontal cortex (PFC) and hippocampus were quickly isolated by hand dissection with a magnifying glass, a spatula, and a thin brush, performed by a qualified researcher. The dissection was based on the histological distinctions described by Paxinos and Watson (Paxinos and Watson 2006). Briefly, the PFC samples comprised medial prefrontal areas, including the prelimbic and infralimbic cortices, corresponding approximately to + 2.2 to + 4.2 mm anterior to bregma. The hippocampal samples were obtained from the dorsal hippocampus, spanning approximately − 2.5 to − 4.5 mm posterior to bregma. After removal of the structures and serum, they were placed in microcentrifuge tubes and stored in a freezer at −70 °C prior to biochemical analysis (*n* = 5 per sex per group (Charan and Kantharia 2013; Ricci et al. 2020).

#### Thiobarbituric acid reactive species (TBARS)

Lipid damage in serum, PFC, and hippocampus was quantified by measuring TBARS levels. TBARS formation during an acid-heating reaction was measured as an oxidative stress index, as previously described (Esterbauer & Cheeseman 1990). Briefly, the samples were mixed with 1 mL of 10% trichloroacetic acid (Vetec, CAS 76-03-9) and 1 mL of 0.67% thiobarbituric acid (Sigma-Aldrich, CAS 504-17-6, Saint Louis, MO, USA) and then heated in a boiling water bath for 15 min. Malondialdehyde (MDA) equivalents were determined at an absorbance of 532 nm using 1, 1, 3, 3-tetramethoxypropane (Sigma-Aldrich, CAS 102-52-3, Saint Louis, MO, USA) as an external standard.

#### Protein carbonyls

Oxidative damage to proteins in serum, hippocampus, and PFC was assessed by measuring carbonyl groups via reaction with dinitrophenyl hydrazine (Sigma-Aldrich, Saint Louis, MO, USA), as previously described (Levine et al. 1990). Briefly, proteins were precipitated by adding 20% trichloroacetic acid (Vetec), then redissolved in dinitrophenyl hydrazine, and absorbance was measured at 370 nm. The results are expressed as protein carbonyls per milligram (mg) of protein.

### Statistical analysis

The statistical analysis was conducted using IBM SPSS Statistics 31.0. Inferential analysis was performed at the 5% significance level, corresponding to a 95% confidence level. We tested the normality of the continuous variables using the Shapiro-Wilk Test, and all variables were normally distributed. The analyses were performed using one-way ANOVA followed by Tukey’s post hoc test and paired-samples t-tests to evaluate differences between training and test sessions. The results are expressed as the mean ± standard error of the mean. Statistical significance was considered for* P*-values < 0.05. Figures were made on Microsoft Excel.

## Results

**Fig. 1:**
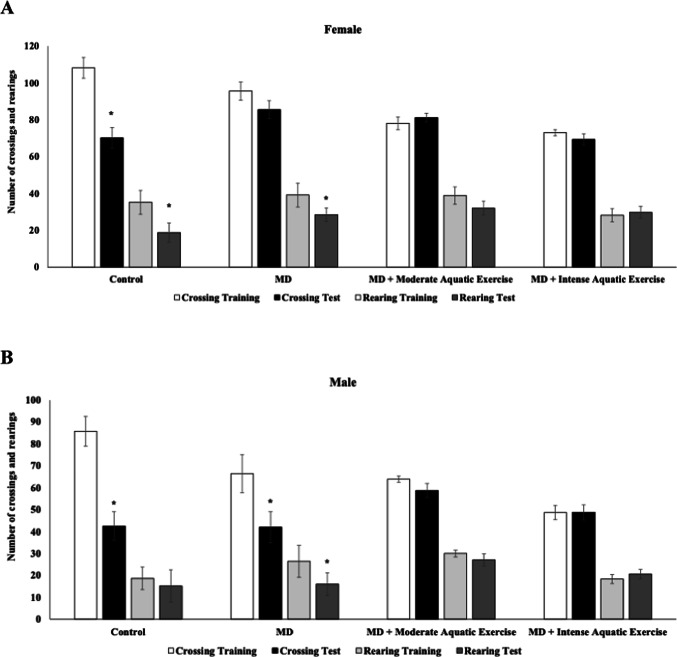
Moderate aquatic exercise or intense aquatic exercise in rats subjected to maternal deprivation (MD) in the open field habituation test. The experimental groups were the control, MD, MD+ moderate aquatic exercise, and MD+ intense aquatic exercise. The number of crossings and rearings by female (**A**) and male (**B**) rats was recorded during the training and test sessions. Values are expressed as mean ± SEM. *p < 0.05 compared to the training session

Figure 1 shows the effects of maternal deprivation and physical exercise protocols on memory habituation. In female rats of the control group, there was a reduction in the number of crossings (t = 7.510, df = 11; *p* < 0.0001, Fig. 1A) and rearings (t = 6.256, df = 11; *p* < 0.0001, Fig. 1A) during the session test (*p* < 0.05, Fig. 1A), consistent with learning acquisition as indicated by the environment recognition test. Females subjected to MD had a reduction in the number of rearing from session tests (t = 3.870, df = 9; *p* = 0.004, Fig. 1A). However, in the number of crossings, no significant difference was found between training and test sessions (*p* = 0.119, Fig. 1A). In the female rats subjected to MD and moderate aquatic exercise or intense aquatic exercise, no significant difference was found between training and test sessions, indicating an impairment in the environment recognition test (*p* > 0.05, Fig. 1A). In male rats of the control group the number of crossings (t = 7.881, df = 10; *p* < 0.0001, Fig. 1B), but not rearings (t = 1.180, df = 10; *p* = 0.265, Fig. 1B), were reduced from session test. In male rats subjected to MD, there was a reduction in the number of crossings (t = 3.890, df = 11; *p* = 0.003) and rearings (t = 3.519, df = 11; *p* = 0.005) from the session test (Fig. 1B). However, in the male rats subjected to MD and moderate aquatic exercise or intense aquatic exercise no significant difference was found between training and test sessions, indicating an impairment in the environment recognition test in stressed male rats subjected to exercise protocols (*p* > 0.05, Fig. 1B).

**Fig. 2:**
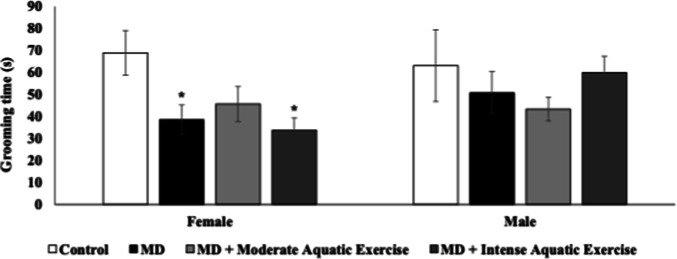
Moderate aquatic exercise or intense aquatic exercise in rats subjected to maternal deprivation (MD) in the grooming time of female and male rats. The experimental groups were the control, MD, MD+ moderate aquatic exercise, and MD+ intense aquatic exercise. The grooming time was recorded in seconds. Values are expressed as mean ± SEM. *p < 0.05 compared to the control group.

Figure 2 illustrates the grooming time in female and male rats subjected to maternal deprivation and physical exercise protocols. The results showed that female rats subjected to MD and MD plus intense aquatic exercise protocols had decreased grooming time compared with the control group (F_(3−38)_ = 4.246, *p* = 0.01, Fig. 2). In male rats, no difference between groups was found in grooming time (F_(3−40)_ = 0.787, *p* = 0.508, Fig. 2).

**Fig. 3:**
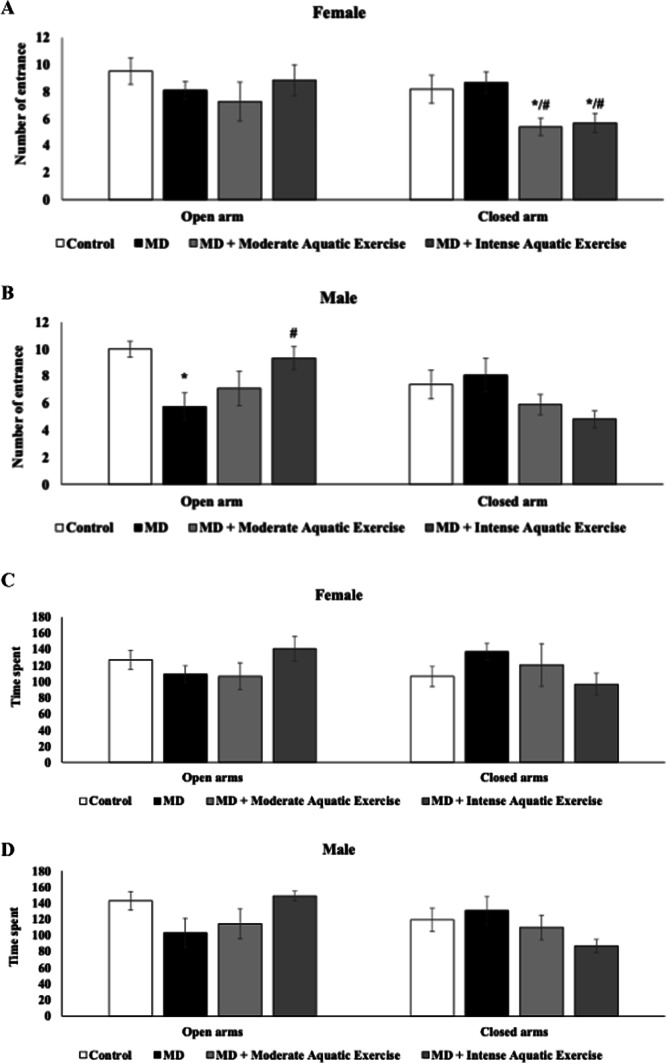
Moderate aquatic exercise or intense aquatic exercise in rats subjected to maternal deprivation (MD) in the number of entrances in open and closed arms of female (**A**) and male (**B**) rats, and time spent in the open and closed arms of female (**C**) and male (**D**) rats. The experimental groups were the control, MD, MD+ moderate aquatic exercise, and MD+ intense aquatic exercise. Values are expressed as mean ± SEM. *p < 0.05 compared to the control group; #*p* < 0.05 compared to the MD group

Figure 3 shows the effects of maternal deprivation and physical exercise protocols on anxious behavior in the elevated plus maze test. In female rats, no difference was reported in the number of entrances for open arms between groups (*p* > 0.05, Fig. 3A). However, in the closed arms, a reduction in the number of entrances was found in the female rats subjected to MD plus moderate aquatic exercise or MD plus intense aquatic exercise compared to the control and MD group (F_(3−40)_ = 3.902, *p* = 0.016, Fig. 3A), indicating an anxiolytic-like effect of the exercise protocols. In the male there was a reduction in the MD group, compared to the control and an increase in the MD plus intense aquatic exercise group, compared to MD in the number of entrances to open arms (F_(3−44)_ = 4.472, *p* = 0.008, Fig. 3B). In addition, in the male rats no difference in the number of entrances to closed arms was reported (F_(3−44)_ = 2.277, *p* = 0.093, Fig. 3B). In the spent time in the open or closed arms no difference was reported in male or female rats (*p* > 0.05, Fig. 3C and D).

**Fig. 4:**
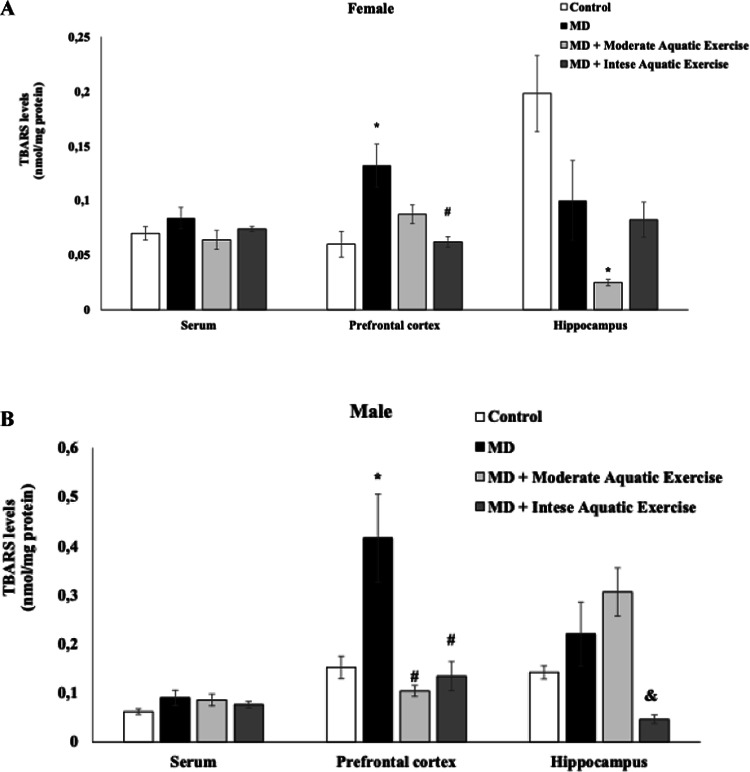
Moderate aquatic exercise or intense aquatic exercise in rats subjected tomaternal deprivation (MD) in the thiobarbituric acid reactive substances (TBARS)levels in the serum, prefrontal cortex, and hippocampus of female (A) and male (B)rats. The experimental groups were the control, MD, MD+ moderate aquaticexercise, and MD+ intense aquatic exercise. Values are expressed as mean ± SEM.*p < 0.05 compared to the control group; #*p* < 0.05 compared to the MD group; &*p*< 0.05 compared to the MD+TAE group

**Fig. 5:**
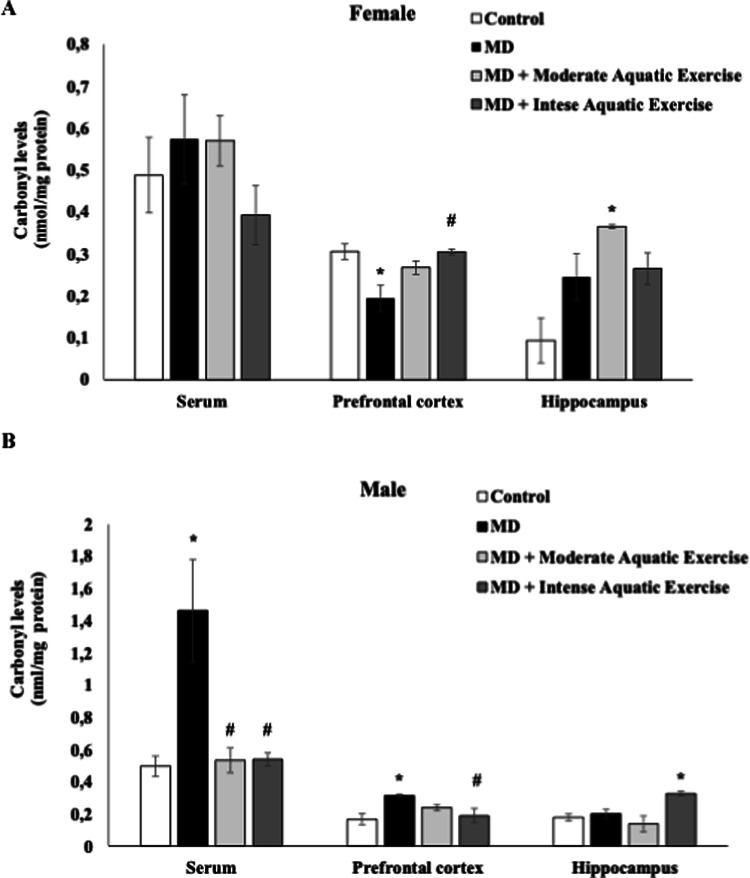
Moderate aquatic exercise or intense aquatic exercise in rats subjected tomaternal deprivation (MD) in the carbonyl levels in the serum, prefrontal cortex,and hippocampus of female (**A**) and male (**B**) rats. The experimental groups were thecontrol, MD, MD+ moderate aquatic exercise, and MD+ intense aquatic exercise.Values are expressed as mean ± SEM. *p < 0.05 compared to the control group; #*p*< 0.05 compared to the MD group.

Oxidative damage to lipids and proteins is described in Figs. 4 and 5, respectively. The TBARS levels were increased in the PFC of female rats subjected to MD, compared to control, and reduced in the MD plus intense aquatic exercise group, compared to the MD group (F_(3−15)_ = 6.996, *p* = 0.004, Fig. 4A). In the hippocampus of female’s rats, it was observed a reduction of TBARS levels in the MD plus moderate aquatic exercise, compared to control (F_(3−14)_ = 6.125, *p* = 0.007, Fig. 4A). In the male rats, TBARS levels were increased in the PFC of MD group, compared to control and reduced in the MD plus intense aquatic exercise and the MD plus moderate aquatic exercise groups, compared to MD group (F_(3−16)_ = 8.682, *p* = 0.001, Fig. 4B). In the male rat’s hippocampus TBARS levels were reduced in the MD plus intense aquatic exercise, compared to MD plus moderate aquatic exercise group (F_(3−16)_ = 7.097, *p* = 0.003, Fig. 4B). In the serum of female and male no difference between groups was found for TBARS levels (*p* > 0.05; Fig. 4A and B).

Carbonyl levels in the PFC of female’s rats were reduced in the MD group, compared to the control and increased in the MD plus intense aquatic exercise group, compared to MD (F _(3−15)_ = 6.873, *p* = 0.004, Fig. 5A). In the hippocampus of female rats, carbonyl levels were increased in the MD plus moderate aquatic exercise group, compared to control (*p* < 0.05; Fig. 5A). In the serum of female rats, no difference was found in the carbonyl levels (*p* > 0.05; Fig. 5A). In the male rats the carbonyl protein levels were increased in the serum of rats subjected to MD, and both exercise protocols decreased carbonyl levels (F _(3−16)_ = 7.722, *p* = 0.002, Fig. 5B). In the PFC carbonyl protein levels were increased in rats subjected to MD, and intense aquatic exercise protocol decreased carbonyl levels (F _(3−16)_ = 4.532, *p* = 0.018, Fig. 5B). However, and intense aquatic exercise protocol increased carbonyl protein levels in the hippocampus of MD rats (F _(3−16)_ = 6.758, *p* = 0.004, Fig. 5B).

## Discussion

The present study showed that male and female rats subjected to early-life stress and intense aquatic exercise or moderate aquatic exercise did not differ significantly when evaluated before the environment recognition test (Fig. 1), indicating that early-life stress combined with exercise protocols did not significantly modify learning acquisition in this task, as assessed by the environment recognition test. When performed at high intensity, physical exercise can impact behavioral and cognitive parameters in several ways, depending on duration, frequency, intensity, and the individual’s physiological and psychological state (Alves et al. 2021; Batrakoulis and Fatouros 2022). In our study, the methodology included high-intensity training and long-duration exercises, which may explain the contradictory results.

One possible explanation for these findings is that prolonged exercise sessions may impose a substantial physiological load, increasing metabolic demand and reactive oxygen species production (Powers and Jackson 2008; Radak et al. 2008, 2014). In addition, exercise-induces activation of the hypothalamic–pituitary–adrenal axis may elevate circulating glucocorticoids such as corticosterone, particularly under conditions of high training volume or intensity (Droste et al. 2007). When training variables are not properly balanced, excessive exercise load may also lead to fatigue-related responses and metabolic stress (Meeusen et al. 2006). Therefore, the beneficial effects of exercise appear to depend on the appropriate manipulation of intensity, duration, and recovery periods (Gomes et al. 2012). The interpretation of behavioral outcomes in females should account for exercise-induced increases in locomotion and exploratory behavior. However, as analyses were conducted within the maternal deprivation context, the effects observed in exercised MD animals likely reflect modulation of stress-related alterations rather than solely improved physical performance.

Figure 2 shows the results of the splash test, which measures anhedonic behavior, indicating that deprived females and MD+ females who engaged in intense aquatic exercise exhibited shorter grooming times, consistent with typical depressive behavior. Females are depressed twice as much as males, and early postnatal adversity may contribute to the psychopathology of depression, especially in vulnerable individuals, triggering anhedonic behavior (Houwing et al. 2019). In turn, advanced aquatic exercise was not able to reverse this behavior.

The elevated plus maze test was used to evaluate anxiety-like behavior. It is important to consider that entering the closed arms represents a natural protective behavior in rodents and, therefore, should not be interpreted as anxiety per se. Instead, anxiety-related responses are more appropriately assessed by the relative exploration of open versus closed arms. According to Fig. 3, rats subjected to MD combined with intense aquatic exercise or MD plus moderate aquatic exercise (females) showed fewer entries into the closed arms when compared with the deprived group, suggesting a shift in the exploratory pattern after the exercise protocols. However, no differences were reported in the time spent in the closed or open arms. Previous studies have reported that animals exposed to stress tend to reduce exploration of the open arms and increase use of the closed arms (Heggelund et al. 2014; Wu et al. 2015).

Differences between our findings and those reported in the literature may also be related to variations in exercise protocols. Although aerobic exercise is generally associated with moderate metabolic demand, the protocol characteristics used in the present study (including session duration, intensity, and workload) may impose a greater physiological challenge, potentially influencing behavioral responses. Previous studies have shown that high-intensity exercise can reduce anxiety and psychological distress in individuals with mental disorders shortly after the exercise session (Heggelund et al. 2014; Wu et al. 2015). In addition, physical exercise has been associated with several psychological benefits, including reductions in anxiety and stress and improvements in self-esteem and cognitive function (Batista and de Oliveira 2015). Therefore, variations in exercise intensity and training load may partly explain the behavioral differences observed across studies.

Figures 4 and 5 present the results of the oxidative stress evaluation in rats subjected to both protocols. Early stress (here represented by maternal deprivation) is related to MDD, affecting behavioral issues and oxidative stress levels in rats (Réus et al. 2023). Studies show that individuals with MDD have elevated levels of TBARS compared to healthy individuals, suggesting an increase in oxidative stress in these individuals (Bengesser et al. 2015; Bilici et al. 2001).

Female rats subjected to MD and intense aquatic exercise showed lower TBARS levels in the PFC than the MD group. In the hippocampus, only females in the MD+ moderate aquatic exercise group showed a decrease in TBARS levels compared to the other groups. In male rats, TBARS levels decreased in the PFC in both exercise groups (MD+ moderate aquatic exercise and MD+ intense aquatic exercise) compared with the MD group. However, in the male hippocampus, TBARS levels increased in the MD+ moderate aquatic exercise group and decreased in the MD+ intense aquatic exercise group compared with the MD group. Although these results appear heterogeneous across brain regions and sexes, they suggest that physical exercise modulated oxidative damage markers associated with maternal deprivation. In the present study, oxidative damage was assessed through TBARS levels, which reflect lipid peroxidation. Previous studies have also demonstrated that long-term exercise can influence redox homeostasis by modulating oxidative and antioxidant systems. For example, Gomez-Cabrera et al. (2008) reported that chronic exercise alters the activity of antioxidant enzymes such as superoxide dismutase and catalase, while Schuch et al. (2016) described the beneficial effects of exercise on oxidative balance and mental health outcomes. Although these studies evaluated different oxidative stress markers and experimental conditions, together they support the notion that exercise can influence redox regulation and potentially contribute to behavioral improvements.

Previous studies have also demonstrated the beneficial effects of aquatic exercise on oxidative balance. For example, Nonato et al. (2016) investigated the effects of an aquatic training protocol in Wistar rats and reported reductions in TBARS levels and increases in superoxide dismutase activity after eight weeks of training, suggesting improved antioxidant defense. However, in contrast to that study, the present work evaluated the effects of different exercise intensities in animals exposed to early-life stress through maternal deprivation. Additionally, our study examined sex-dependent responses and assessed oxidative stress markers in distinct brain regions, including the PFC and hippocampus. These differences in experimental design provide further insight into how exercise intensity may influence oxidative stress regulation and behavioral outcomes under stress-related conditions.

Individuals with MDD have been reported to exhibit elevated levels of protein carbonyls, reflecting increased oxidative damage that may contribute to cognitive and emotional dysfunctions (Ignácio, 2017a). Similarly, Qin et al. (2024) demonstrated that rodents exposed to stress showed increased indoleamine 2, 3-dioxygenase (IDO) activity and elevated carbonyl protein levels in the prefrontal cortex, which were associated with depressive-like behaviors. However, differences between the studies should be considered when interpreting these findings. Qin et al. focused on a specific stress model and evaluated only the PFC, whereas the present study investigated both the PFC and hippocampus and included male and female animals subjected to early-life stress through maternal deprivation combined with exercise interventions. In the present study, protein carbonyl levels varied according to sex, brain region, and exercise protocol. Higher levels were observed in females, including those who engaged in physical exercise, whereas in males, exercise was generally associated with lower carbonyl levels, except in the MD + intense aquatic exercise group, which showed increased carbonyl levels in the hippocampus. These findings suggest that, although stress-related oxidative damage is consistently observed across studies, its modulation may depend on experimental conditions such as stress model, sex, brain region, and exercise load, highlighting the complex and context-dependent nature of oxidative responses.

The intensity and duration of physical exercise can lead to adverse effects when performed at high intensity, which may be a limitation of the present study. High-intensity physical exercise can affect behavioral and cognitive parameters in several ways, depending on factors such as duration, frequency, intensity, and the individual’s physiological and psychological state. Excessive physical exercise increases ROS production. It promotes systemic inflammation, impairs synaptic plasticity and cognitive performance, and induces muscle damage, resulting in poor exercise performance (Cruzat et al. 2007; Thirupathi et al. 2021).

In one study, only the high-intensity exercise (18 m/min, EX18, 30 min/day for 7 consecutive days) improved memory performance by increasing brain-derived neurotrophic factor (BDNF) levels in the hippocampus and PFC. Therefore, the study reported that the improvement in memory associated with BDNF depends on exercise intensity (Cefis et al. 2019). It has been shown that exercise initiated in adolescence does not continually improve memory; however, it induces an increase in the complexity of neurites in doublecortin cells, an essential process for synaptic integration, compared with exercise initiated later in life (O’Leary et al. 2019). Another study with rats investigated the effects of high-intensity interval training (HIIT) on oxidative stress, BDNF levels, and inflammatory mediators in the hippocampus. After six weeks of high-intensity exercise (at ~ 85–100% of VO2max), the rodents showed a significant reduction in oxidative damage, increases in enzymatic (e.g., superoxide dismutase) and non-enzymatic antioxidant defenses, and increased BDNF levels in the hippocampus (Freitas et al. 2018). These findings suggest that HIIT may improve brain health by modulating oxidative stress and promoting neuroplasticity through increased BDNF.

Previous studies indicate that exercise can exert both antioxidant and pro-oxidant effects depending on its intensity and duration (Gomez-Cabrera et al. 2008; Radak et al. 2016), and that improvements in oxidative markers do not necessarily parallel cognitive outcomes (Cechetti et al. 2008). Additionally, heterogeneous responses across oxidative parameters have been reported (Nonato et al. 2016), reinforcing the complexity of these interactions.

The effects of exercise depend closely on its intensity and duration, which may help explain the variability in outcomes related to MDD, anhedonia, and cognition. While moderate exercise is generally associated with cognitive benefits, higher intensities or prolonged exposure may induce physiological stress and impair memory performance. In the present study, although the protocol improved anxiety-like behavior, the observed cognitive effects may be partly related to the specific exercise parameters, highlighting the need for careful modulation of exercise dose in future studies.

The inclusion of both sexes allowed the identification of sex-dependent responses to maternal deprivation and exercise interventions. Previous studies indicate that males and females differ in their physiological and behavioral responses to stress, partly due to hormonal influences and neuroendocrine regulation (Bangasser and Valentino 2014; Beery and Zucker 2011). Estrogens, for example, have been reported to exert antioxidant and neuroprotective effects, which may influence oxidative stress regulation and neuronal plasticity (Arevalo et al. 2010; Gomez-Cabrera et al. 2006). In addition, sex differences in hypothalamic–pituitary–adrenal axis activity may contribute to distinct behavioral and neurobiological outcomes following early-life stress (Bangasser and Valentino 2014). These mechanisms may partly explain the variability observed between males and females in oxidative stress markers and behavioral responses in the present study.

Although the present study provides relevant insights into the effects of early-life stress and exercise on behavioral and oxidative parameters, some limitations should be considered. The high intensity and prolonged duration of the exercise protocols may have influenced the outcomes in unexpected ways, potentially masking beneficial effects in certain groups. Another limitation of the present study is the absence of an exercise-only group, which restricts the ability to distinguish the isolated effects of exercise from its interaction with early-life stress; therefore, future studies should include non-stressed exercised groups to better disentangle these effects. Additionally, the variability observed between sexes and brain regions suggests that responses to stress and exercise are complex and multifactorial. Despite these limitations, the study offers valuable contributions to understanding the interaction between physical exercise, early adversity, and neurobiological outcomes.

## Conclusion

The evidence from this study suggests that the maternal deprivation protocol induces anhedonic behavior in females and that advanced exercise maintains this behavior. However, both intense and moderate exercise decreased anxiety in females and increased anxious behavior induced by maternal deprivation in males, but only advanced exercise reversed this effect. In memory, maternal deprivation-induced deficits were observed in females and males across both exercises. On the other hand, both intense and moderate exercise reduced lipid and protein damage caused by MD, but these effects were brain area- and sex-dependent.

Further studies are needed to identify the ideal exercise protocol for MDD that can standardize the beneficial effects on anhedonia, anxiety, and cognition. In addition, further studies to evaluate the behavioral and neurochemical effects of early stress are of paramount importance. Another challenge is to identify the influence of sex on the neurobiology of MDD and physical exercise practice, which should be examined more closely in research.

## Data Availability

No datasets were generated or analysed during the current study.
